# Prevalence and Genotyping of *Cryptosporidium parvum* in Gastrointestinal Cancer Patients

**DOI:** 10.7150/jca.42393

**Published:** 2020-03-05

**Authors:** Nan Zhang, Xiuyan Yu, Hongbo Zhang, Lianying Cui, Xiaoou Li, Xiaowei Zhang, Pengtao Gong, Jianhua Li, Ziyi Li, Xiaocen Wang, Xin Li, Ting Li, Xiaofeng Liu, Yanhui Yu, Xichen Zhang

**Affiliations:** 1 The First Hospital, Key Laboratory of Zoonosis Research by Ministry of Education, Institute of Zoonosis, Jilin University, Changchun 130021, China.; 2 Jilin Cancer Hospital, Changchun 130021, China.; 3 Key Laboratory of Zoonosis Research by Ministry of Education, Institute of Zoonosis, College of Veterinary Medicine, Jilin University, Changchun 130062, China.; 4 Clinical Lab, The Second Hospital, Jilin University, Changchun 130021, China.

**Keywords:** *C. parvum*, Epidemiology, Colorectal cancer, Liver cancer, Esophageal cancer, Small intestine cancer

## Abstract

Gastrointestinal cancers are the most commonly occurring malignancies which contributing to over 1/5 of cancer incidences worldwide. Increasing evidences have shown that *Cryptosporidium* spp., an apicomplexan protozoan, is highly associated with gastrointestinal cancers. However, the prevalence of *Cryptosporidium* spp. infections among gastrointestinal cancer patients in China has not been estimated yet. We here performed a case-control study to evaluate the occurrences of *Cryptosporidium* spp. in patients with digestive malignancies before chemotherapy and in control population. Nested PCR amplifying *18S* rRNA gene was used to detect the presence of *Cryptosporidium* spp. in each fecal sample. The results herein confirmed the correlation of *Cryptosporidium* spp. infection with colorectal and liver cancers, while first identified the high frequencies of *Cryptosporidium* spp. in esophageal cancer and small intestine cancer. The infection rates of *Cryptosporidium* spp. in colorectal, esophageal, liver and small intestine cancers were 17.24% (20/116, *P*<0.001), 6.25% (1/16, *P*=0.029), 14.29% (1/7, *P*<0.001) and 40% (2/5, *P*<0.001), respectively. In addition, molecular characterization indicated that all the *Cryptosporidium* spp. obtained were *Cryptosporidium parvum* (*C. parvum*), and the *18S* rRNA sequences were identical to the reference sequences isolated from cattle, suggesting potential zoonotic transmission. Furthermore, subtyping analyses revealed that IIaA15G2R1 and IIaA15G2R2 were the predominant subtypes in colorectal cancer, while IIaA13G2R2 subtype was first named and identified in colorectal and liver cancers. Taken together, for the first time, the prevalence of *Cryptosporidium* spp. infections in digestive cancer patients has been estimated among Chinese. Our results indicated that *C. parvum* were highly associated with gastrointestinal cancers, supporting that cryptosporidiosis could be a potential risk factor for these diseases.

## Introduction

Gastrointestinal cancers are the most commonly diagnosed cancers and the leading cause of cancer deaths with over 30% for mortality in 2018 1,2. Genetic or epigenetic defects are considered to be the causative reasons for a variety of digestive malignancies. Nevertheless, infectious agents, which contributed to about 20% of the cancer incidence worldwide, also cannot be ignored 3. Thus far, a number of infectious agents have been identified as causative factors or contributors of various gastrointestinal cancers. The well-known infectious agents, Hepatitis B and C viruses; are the leading risk factors for liver cancer 4. *Helicobacter pylori* infection is a major cause of gastric cancer 4. In addition, some parasites, such as *Opisthorchis viverrini* and* Clonorchis sinensis*, are also considered to play a crucial role in digestive malignancies 3.

*Cryptosporidium* spp., belongs to apicomplexa protozoan, is a common and important zoonotic pathogen. It infects a wide range of hosts including humans and mainly inhabits the gastrointestinal and respiratory epithelium of vertebrates 5. *Cryptosporidium* infection is a primary etiology of diarrhea in humans. Its secondary infection in AIDS (Acquired Immune Deficiency Syndrome), tumor and organ transplant patients is one of the major causes of death of these patients who have suppressed immune system 6,7. So far, more than 30 species and 70 subtypes of *Cryptosporidium* have been identified 7,8. Among them, *Cryptosporidium hominis (C. hominis)* and *Cryptosporidium parvum (C. parvum*) are the dominant pathogenic species responsible for most of the infection cases in humans 5. *Cryptosporidium* is transmitted by fecal-oral route, mainly through contaminated food or water. It can also be transmitted to humans through direct contact with infected persons. In spite of the severe threat of *Cryptosporidium* infection to immunocompetent and immunocompromised individuals, there is still no ideal prevention and treatment method of cryptosporidiosis till now.

*Cryptosporidium* spp. infections have been suggested to be associated with several cancers, such as blood cancer, colorectal cancer and liver cancer 5. As early as 1988, it has been suspected that *Cryptosporidium* spp. infection may be correlated with colon adenocarcinoma 9. After that, the occurrences of *Cryptosporidium* spp. infections in colorectal cancer patients have been evaluated in different populations. In 2007, the first epidemiological study in Poland showed that 10 out of 55 (18%) patients with diagnosed colorectal cancer had cryptosporidiosis, and the infection rate was even higher in those patients with diarrhea symptom (43.5%, 10/55) 10. Another two reports also conducted in Poland showed similar infection rates (12.6%, 11/87 and 13%, 14/108) 11,12. Recently, Osman M. et al. (2017) performed a systematic analysis among Lebanese patients which indicated that *Cryptosporidium* spp. infections were more prevalent in patients with colon adenocarcinoma (21%, 15/72) than that in patients without digestive cancers but with digestive symptoms (7%, 9/125) 13. This study also identified *C. hominis* and* C. parvum* as the causative species of *Cryptosporidium* spp. Infections in colorectal cancer patients. In addition, a study in Saudi showed high frequencies of *Cryptosporidium* spp. in both colorectal cancer (50%, 6/12) and liver cancer (50%, 2/4) 14. The high association between cryptosporidiosis and hepatocellular carcinoma (32%, 16/32) was also suggested by another study in Egypt 15. Nonetheless, the infection rates of *Cryptosporidium* spp. in other digestive cancers have not been determined yet.

So far, the occurrences of *Cryptosporidium* spp. in China have been evaluated among diarrheal outpatients, AIDS patients and children 16-20. However, *Cryptosporidium* spp. infections in patients with gastrointestinal cancers have not been investigated in China. Thus, the aim of this study was to estimate the prevalence and characterize the species and genotypes/subtypes of *Cryptosporidium* spp. in gastrointestinal cancer patients in Jilin province, China.

## Materials and Methods

### Ethics Statement

Ethical approval of this study was obtained from the Ethical Committee of Jilin Cancer Hospital and the Second Hospital of Jilin University (Reference no.: 201712-047). The purpose of the study was orally explained to all the participants, and the fecal samples were collected with their approval.

### Study populations

This study included 195 patients with diagnosed gastrointestinal cancers and without chemotherapy at Jilin Cancer Hospital and the Second Hospital of Jilin University during January-July, 2018. The cancer cases were determined based on imaging (B ultrasound; computed tomography, CT; nuclear magnetic resonance, NMR) and pathological diagnosis (gold standard). This group consisted of 125 males and 70 females with the average age of 59 years. We also recruited a control group of 141 individuals without diagnosis and history of cancer. The control population was confirmed by health examination in the hospital. This group included 70 males and 71 females and has an average age of 60 years. The detailed information of cancer patients and control population were shown in Table S1 and S2. We have previously used the same samples to detect the presence of *Pentatrichomonas hominis* in gastrointestinal cancer patients 21.

### Sample collection and DNA extraction

The fresh fecal samples from 336 individuals were collected in empty containers and were cooled with ice during transport to the lab. All of the samples were stored at -20 °C until DNA extraction. In order to avoid the risk of cross-contamination, standard procedures for cleaning workspaces and instruments were applied. In addition, internal reference controls were set up at the same time.

Genomic DNA was extracted from each fecal sample using the Fecal DNA Rapid Extraction Kit (TIANGEN, Beijing, China) according to the manufacturer's instructions. The purified DNA samples were stored at -20 °C until PCR analysis.

### PCR amplification

The presence of *Cryptosporidium* spp. in the fecal samples were detected via nested PCR amplifying ~587 bp fragment of the *18S* rRNA gene. The primer sequences and PCR conditions were used as previously described 22. The positive samples were further characterized for the subtypes of *C. parvum* by nested PCR amplifying gp60 sequences as previously described 23. DNA extracted from *C. parvum* (IIaA15G2R1) was used as positive control, while ddH_2_O served as negative control.

### Sequence analysis and genetic characterization of *Cryptosporidium* spp. isolates

The PCR products with positive amplifications were purified and sequenced. Then, the obtained sequences were subjected to BLAST website (https://blast.ncbi.nlm.nih.gov/Blast.cgi) to align with the reference sequences to determine the species and subtypes of *Cryptosporidium* spp. isolates. The novel nucleotide sequences were deposited in the GenBank database under the accession numbers MN704639-41 and MN747318-20.

### Statistical analysis

Data analyses were performed using SPSS software (version 20.0, IBM, Armonk, NY, USA). The differences between different groups were assessed by Pearson chi-square (χ^2^) or Fisher's exact test where indicated. All tests were two-sided, and *P* <0.05 was considered statistically significant.

## Results

In total, *Cryptosporidium* spp. was detected in 26 out of 195 patients with diagnosed gastrointestinal cancers (13.33%). Conversely, none of the control population has *Cryptosporidium* spp. infection (0/141; Table 1). The prevalence of *Cryptosporidium* spp. in gastrointestinal cancer patients was significantly higher than that in controls (*χ*^2^=13.904, df=1, *P*<0.001). In detail, the infection rates of* Cryptosporidium* spp. in colorectal, gastric, esophageal, liver and small intestine cancers were 17.24% (20/116), 4% (2/51), 6.25% (1/16), 14.29% (1/7) and 40% (2/5), respectively (Table 1). Except for gastric cancer (*P*=0.121), all the gastrointestinal cancers were significantly associated with *Cryptosporidium* spp. infections (Table 1).

The age of gastrointestinal cancer patients ranged between 31-87 years with the average age of 59 years. *Cryptosporidium* spp. was found in all the age groups, but there was no obvious difference between different groups (*P*=0.972; Table 2). The infection rate of *Cryptosporidium* spp. was nearly 2-fold higher in males than that in females (16% vs 8.57%; Table 2). However, Since we collected nearly twice as many fecal samples from men as from women (125 vs. 70, Table S1), it is possible that the higher frequency of *Cryptosporidium* spp. infection in males than that in females was due to the larger sample size of males. Moreover, a slightly high frequency of *Cryptosporidium* spp. was observed in patients from urban areas (15.04%) than that from rural areas (10.98%; Table 2). However, no statistical significant difference was found between any of these groups. Similar tendencies of the infection rates were also observed in patients with colorectal cancer (Table S3). But the infection rate in males with colorectal cancer (22.39%) was twice that in females (10.2%), and this was statistical significance.

To determine the species for *Cryptosporidium* spp. infections in gastrointestinal cancer patients, nested PCR was used to detect the *18S* rRNA gene. The results indicated that all of the *Cryptosporidium* spp. identified were *C. parvum*. Of the 26 sequences obtained, 24 sequences were 100% identity to the reference sequences (GenBank ID: JX886768.1, JX886767.1, JX886766.1, JX886765.1, FJ752165.1, Table S4), while two sequences have single nucleotide polymorphisms (SNPs) and were assigned as novel types (Table 3).

Although the prevalence of *Cryptosporidium* spp. has been documented in several digestive cancers, the subtypes have been rarely identified. Here, we used nested PCR amplifying gp60 gene to determine the subtypes of *C. parvum* in gastrointestinal cancer patients. Genetic analyses identified that of 26 isolates obtained, twelve were typed as IIaA15G2R1 and twelve were types as IIaA15G2R2 (Table 4). In addition, we found that another two sequences were homologous to IIaA15G2R1 subtype with the 142 position T substituted by A. According to the nomenclature of *Cryptosporidium* spp., we defined this two novel sequences as IIaA13G2R2 (GenBank ID: MN747320) 24. IIaA13G2R2 was only observed in colorectal and liver cancers, whereas IIaA15G2R1 and IIaA15G2R2 were found in colorectal, gastric and small intestine cancers. In addition, IIaA15G2R2 subtype was also observed in esophageal cancer. These results further confirmed that *C. parvum* was responsible for *Cryptosporidium* spp. infections in all gastrointestinal cancer patients, and suggested the potential zoonotic transmission.

## Discussion

*Cryptosporidium* spp. infection is a severe threat to human health. It is one of the major causes of diarrhea and can be life-threatening among immunocompromised patients. To our knowledge, the present study is the first report performed in China comparing the prevalence of *Cryptosporidium* spp. infections between gastrointestinal cancer patients and people without digestive symptoms. The results herein indicated that *Cryptosporidium* spp. was strongly associated with gastrointestinal cancers, and *C. parvum* was responsible for the infections in digestive cancer patients. Except gastric cancer, all the studied cancer types showed statistically significant correlation with *C. parvum* infection, indicating that it could be a potential etiological factor for these cancers.

The associations of *Cryptosporidium* spp. Infections with digestive cancers have been described in different populations. Several epidemiological studies demonstrated that colorectal cancer is highly correlated with *Cryptosporidium* spp. 10-14. In consistence, we here found that 17.24% patients with colorectal cancer had *C. parvum* infections, which was significantly higher than control population. This infection rate was similar with the previous reports in colorectal cancer patients: 18%, 12.6% and 13% in Poland, and 21% in Lebanon. A link has also been established between cryptosporidiosis and liver cancer 14,15,25. This was also true in our study which showed that *C. parvum* infection was significantly higher in liver cancer patients than that in control population. In addition, we observed 2 out of 51 patients with gastric cancer had *C. parvum*. However, statistical analysis did not reveal the significant correlation between gastric cancer and *C. parvum* infection. This is consistent with the previous study among Lebanese which found none of the gastric cancer patients had *Cryptosporidium* spp. infection 13. Furthermore, for the first time, our study identified the high occurrences of *C. parvum* in patients with esophageal cancer (6.25%, 1/16) and small intestine cancer (40%, 2/5). However, due to the small sample size, further studies with a large collection of samples and in different populations are needed to reach the definitive conclusions of the correlation between *Cryptosporidium* spp. infection and these two cancers.

In fact, the ability of* Cryptosporidium* spp. in the induction of digestive malignancies has been demonstrated in rodent models. Several studies showed that animal-derived *C. parvum* can induce the generation of colorectal, stomach and small intestine tumors using dexamethasone treated SCID (severe combined immunodeficiency) mice 26-29. Also, *C. parvum* of human origin can cause gastrointestinal and biliary adenocarcinoma in SCID mice 30. In contrast, another species *C. muris* did not induce the gastrointestinal cell transformation in the same experimental model 27, suggesting the unique role of *C. parvum* in the development of digestive cancers.

Till now, more than 30 species of *Cryptosporidium* have been identified. *C. parvum* and *C. hominis* account for most of *Cryptosporidium* spp. infections in humans, while *C. meleagridis*, *C. felis*, *C. canis*, *C. muris*,* C. suis* and *C. andersoni* are occasionally observed in humans 7,31. Previous molecular analyses indicated that *C. hominis* and *C. parvum* species were responsible for *Cryptosporidium* infections in colorectal cancer patients 13. In the present study, we found that all the *Cryptosporidium* spp. identified in gastrointestinal cancer patients were *C. parvum*. *C. parvum* is one of the major zoonotic species with a wide range of hosts including humans, cattle and horses 7. BLAST analyses indicated that most of the *18S* rRNA sequences obtained were 100% identity with the GenBank reference sequences: JX886768.1, JX886767.1, JX886766.1, JX886765.1 and FJ752165.1 (Table S4), which were all isolated from cattle, suggesting the potential zoonotic transmission. In addition, two novel sequences were obtained which displayed SNPs when compared with the above reference sequences (Table 3).

Previously, IIaA15G2R1 (one case) and IIcA5G3 (two cases) subtypes in colorectal cancer, and IIaA16G2R1 (one case) and IIaA17G2R1 (22 cases) subtypes in paediatric oncology have been identified 5,23,32. However, the subtypes responsible for *C. parvum* infections in other gastrointestinal cancers have not been determined yet. We here identified that IIaA15G2R1, IIaA15G2R2 and IIaA13G2R2 were the subtypes responsible for *C. parvum* infections in gastrointestinal cancers. Noticeably, two novel sequences with 13 TCA (A), 2 TCG (G) and 2 ACATCA (R) repeats were first identified and defined as IIaA13G2R2 subtype and were observed in both colorectal and liver cancers. For the prevalence of subtypes in colorectal cancer, IIaA15G2R1 and IIaA15G2R2 were the predominant subtypes and were detected in almost equal frequencies, whereas IIaA13G2R2 was only found in one case. Additionally, both IIaA15G2R1 and IIaA15G2R2 were observed in small intestine cancer, but only IIaA15G2R2 has been found in esophageal cancer. It has been demonstrated that subtype families of *C. parvum* have different host specificity. IIa family is mainly found in cattle and is responsible for zoonotic cryptosporidiosis 24. IIaA15G2R1 is the most prevalent subtype for *C. parvum* worldwide and is mostly found in calves 7, while IIaA15G2R2 has been identified in calves and humans in the United States 33. Thus, these results further supported a potential zoonotic transmission of cryptosporidiosis in digestive cancer patients. Nevertheless, the transmission sources and hosts of IIaA13G2R2 subtype need to be further investigated.

The pathological mechanism underlying *Cryptosporidium* spp. infections in the induction of digestive cancers remains unclear. However, available data suggested that *Cryptosporidium* spp., for its survival and transmission, can interfere with the cell signaling pathways and impact the gene expressions in host cells, thus, may secondarily promote the generation of gastrointestinal cancers. It has been shown that *C. parvum* can inhibit the apoptosis of infected biliary epithelia through activating the NF-κB signaling pathway 34. A microarray analysis also revealed that *C. parvum* infection affected a large number of apoptosis gene expressions with upregulation of antiapoptotic genes and downregulation of proapoptotic genes at early stage 35. Resistance to apoptosis is one of the hallmarks of cancer development 36. Thus, *C. parvum* may induce the gastrointestinal cancers via preventing infected cells from cell death. Additionally, Benamrouz S. et al. found that *C. parvum* infections can alter the Wnt signaling pathway components and is able to modulate the cytoskeleton network in infected cells. They also showed that the changes of these biological processes were involved in the progression of ileo-caecal oncogenesis 37. However, further work is still needed to clarify the molecular mechanisms of gastrointestinal epithelia transformation induced by *Cryptosporidium*.

In conclusion, the present study, for the first time, estimated the prevalence of *Cryptosporidium* spp. and characterized its species and subtypes in gastrointestinal cancer patients in China. Nevertheless, the causal relationship of *C. parvum* infections in the development of gastrointestinal cancers needs to be further elucidated.

## Supplementary Material

Supplementary tables.

## Figures and Tables

**Table 1:** Frequencies of *Cryptosporidium* spp. in gastrointestinal cancer patients and controls

Groups	No. examined	No. positive	%	*Χ^2^/df/P-value*
Controls	141	0	0	Reference
Gastrointestinal cancers	195	26	13.33	13.904/1/ <0.001
Cancer types				
Colorectal cancer	116	20	17.24	18.579/1/<0.001
Gastric cancer	51	2	4	-/-/0.121^#^
Esophageal cancer	16	1	6.25	-/-/0.029^#^
Liver cancer	7	1	14.29	15.054/1/<0.001
Small intestinal cancer	5	2	40	50/1/ <0.001

#Fisher's exact test.

**Table 2:** Frequencies of *Cryptosporidium* spp. in gastrointestinal cancer patients by age, sex and residence (n=195)

Characteristic	No. examined	No. positive	%	*Χ^2^/df/P-value*
**Age (years)**				
≤50	44	6	13.64	0.058/2/0.972
51-60	61	8	13.11	
>60	90	12	13.33	
**Sex**				
Male	125	20	16	2.24/1/0.134
Female	70	6	8.57	
**Residence**				
Urban	113	17	15.04	0.707/1/0.4
Rural	82	9	10.98	

**Table 3:** SNPs of the *18S* rRNA gene of *C. parvum* identified in this study

Groups	GenBank ID	Isolate No.	Type	SNPs					
				369^#^	390^#^	398^#^	410^#^	484^#^	499^#^
Colorectal cancer	MN704639	18	CpCH1*	G	G	G	C	G	G
	MN704640	1	CpCH2	G	G	G	G	G	G
	MN704641	1	CpCH3	T	A	T	G	T	A
Gastric cancer	MN704639	2	CpCH1	G	G	G	C	G	G
Esophageal cancer	MN704639	1	CpCH1	G	G	G	C	G	G
Liver cancer	MN704639	1	CpCH1	G	G	G	C	G	G
Small intestine cancer	MN704639	2	CpCH1	G	G	G	C	G	G

*CpCH: *C*. *parvum* Changchun Human. ^#^means the nucleotide position.

**Table 4:** Molecular characterization of *C. parvum* identified in this study

GenBank ID	Type	Species	Genotype	Isolate no.	Cancer type (number of isolates)	Reference sequence	SNPs
MN747318	CPgp1	*C. parvum*	IIaA15G2R1	12	Colorectal (10), Gastric (1), Small intestine (1)	AY262034	-
MN747319	CPgp2	*C. parvum*	IIaA15G2R2	12	Colorectal (9), Gastric (1), Small intestine (1), Esophageal (1)	DQ192501	-
MN747320	CPgp3	*C. parvum*	IIaA13G2R2	2	Colorectal (1), Liver (1)	AY262034	T142A

## Abbreviations

AIDS: Acquired Immune Deficiency Syndrome
CT: computed tomography
NMR: nuclear magnetic resonance
SCID: severe combined immunodeficiency
SNPs: single nucleotide polymorphisms
