# Supplementary material for: Prevalence and Genotyping of Cryptosporidium parvum in Gastrointestinal Cancer Patients
Source: J Cancer. 2020 Mar 5;11(11):3334–9. doi: 10.7150/jca.42393 (PMC7097938; doi:10.7150/jca.42393)
Supplement: Supplementary file 1 — Supplementary tables. [file jcav11p3334s1.pdf]

Table S1. Characteristics of the control population and patients with gastrointestinal cancers.

Characteristic	Control population<br>No. examined (%)<br>n=141	Gastrointestinal cancer patients<br>No. examined (%)<br>n=195	X^2/*df*/*P-value*
**Age (years)**			
≤50	22 (15.6)	44 (22.56)	2.125/2/0.346
51-60	41 (29.08)	61 (31.28)	
>60	78 (55.32)	90 (46.15)	
**Sex**			
Male	70 (49.65)	125 (64.1)	3.998/1/0.046
Female	71 (50.35)	70 (35.9)	
**Residence**			
Urban	90 (63.83)	113 (57.95)	0.757/1/0.384
Rural	51 (36.17)	82 (42.05)	

Table S2. Characteristics and *Cryptosporidium* spp. infection information of 195 gastrointestinal cancer patients

	Sex	Age	Residence	Cancer types	*Cryptosporidium* spp. infection	*Cryptosporidium* spp. species	*Cryptosporidium* spp. genotype
1	Male	63	Urban	Colorectal cancer	Positive	*C. parvum*	IIaA15G2R1
2	Male	51	Urban	Colorectal cancer	Positive	*C. parvum*	IIaA15G2R2
3	Male	55	Urban	Colorectal cancer	Positive	*C. parvum*	IIaA15G2R2
4	Male	40	Urban	Colorectal cancer	Positive	*C. parvum*	IIaA15G2R2
5	Male	68	Urban	Colorectal cancer	Positive	*C. parvum*	IIaA15G2R1
6	Male	78	Urban	Colorectal cancer	Positive	*C. parvum*	IIaA15G2R2
7	Male	53	Urban	Colorectal cancer	Positive	*C. parvum*	IIaA15G2R1
8	Male	61	Urban	Colorectal cancer	Positive	*C. parvum*	IIaA15G2R2
9	Male	69	Urban	Colorectal cancer	Positive	*C. parvum*	IIaA15G2R2
10	Male	35	Urban	Colorectal cancer	Positive	*C. parvum*	IIaA15G2R2
11	Male	62	Rural	Colorectal cancer	Positive	*C. parvum*	IIaA15G2R1
12	Male	62	Rural	Colorectal cancer	Positive	*C. parvum*	IIaA15G2R1
13	Male	80	Rural	Colorectal cancer	Positive	*C. parvum*	IIaA15G2R1
14	Male	46	Rural	Colorectal cancer	Positive	*C. parvum*	IIaA15G2R1
15	Male	63	Rural	Colorectal cancer	Positive	*C. parvum*	IIaA15G2R1
16	Female	66	Urban	Colorectal cancer	Positive	*C. parvum*	IIaA15G2R1
17	Female	48	Urban	Colorectal cancer	Positive	*C. parvum*	IIaA15G2R1
18	Female	61	Rural	Colorectal cancer	Positive	*C. parvum*	IIaA15G2R2
19	Female	59	Rural	Colorectal cancer	Positive	*C. parvum*	IIaA15G2R2
20	Female	48	Rural	Colorectal cancer	Positive	*C. parvum*	IIaA15G2R2
21	Male	65	Urban	Colorectal cancer	Negative	-	-
22	Male	52	Urban	Colorectal cancer	Negative	-	-
23	Male	36	Urban	Colorectal cancer	Negative	-	-

24	Male	46	Urban	Colorectal cancer	Negative	-	-
25	Male	65	Urban	Colorectal cancer	Negative	-	-
26	Male	50	Urban	Colorectal cancer	Negative	-	-
27	Male	57	Urban	Colorectal cancer	Negative	-	-
28	Male	58	Urban	Colorectal cancer	Negative	-	-
29	Male	59	Urban	Colorectal cancer	Negative	-	-
30	Male	33	Urban	Colorectal cancer	Negative	-	-
31	Male	52	Urban	Colorectal cancer	Negative	-	-
32	Male	60	Urban	Colorectal cancer	Negative	-	-
33	Male	79	Urban	Colorectal cancer	Negative	-	-
34	Male	48	Urban	Colorectal cancer	Negative	-	-
35	Male	49	Urban	Colorectal cancer	Negative	-	-
36	Male	59	Urban	Colorectal cancer	Negative	-	-
37	Male	61	Urban	Colorectal cancer	Negative	-	-
38	Male	45	Urban	Colorectal cancer	Negative	-	-
39	Male	48	Urban	Colorectal cancer	Negative	-	-
40	Male	66	Urban	Colorectal cancer	Negative	-	-
41	Male	43	Urban	Colorectal cancer	Negative	-	-
42	Male	55	Urban	Colorectal cancer	Negative	-	-
43	Male	63	Urban	Colorectal cancer	Negative	-	-
44	Male	74	Urban	Colorectal cancer	Negative	-	-
45	Male	63	Urban	Colorectal cancer	Negative	-	-
46	Male	70	Urban	Colorectal cancer	Negative	-	-
47	Male	59	Urban	Colorectal cancer	Negative	-	-
48	Male	60	Urban	Colorectal cancer	Negative	-	-
49	Male	68	Urban	Colorectal cancer	Negative	-	-

50	Male	54	Rural	Colorectal cancer	Negative	-	-
51	Male	57	Rural	Colorectal cancer	Negative	-	-
52	Male	66	Rural	Colorectal cancer	Negative	-	-
53	Male	54	Rural	Colorectal cancer	Negative	-	-
54	Male	66	Rural	Colorectal cancer	Negative	-	-
55	Male	57	Rural	Colorectal cancer	Negative	-	-
56	Male	61	Rural	Colorectal cancer	Negative	-	-
57	Male	56	Rural	Colorectal cancer	Negative	-	-
58	Male	68	Rural	Colorectal cancer	Negative	-	-
59	Male	64	Rural	Colorectal cancer	Negative	-	-
60	Male	50	Rural	Colorectal cancer	Negative	-	-
61	Male	68	Rural	Colorectal cancer	Negative	-	-
62	Male	61	Rural	Colorectal cancer	Negative	-	-
63	Male	61	Rural	Colorectal cancer	Negative	-	-
64	Male	65	Rural	Colorectal cancer	Negative	-	-
65	Male	61	Rural	Colorectal cancer	Negative	-	-
66	Male	53	Rural	Colorectal cancer	Negative	-	-
67	Male	50	Rural	Colorectal cancer	Negative	-	-
68	Male	58	Rural	Colorectal cancer	Negative	-	-
69	Male	54	Urban	Colorectal cancer	Negative	-	-
70	Male	59	Rural	Colorectal cancer	Negative	-	-
71	Male	45	Rural	Colorectal cancer	Negative	-	-
72	Male	52	Rural	Colorectal cancer	Negative	-	-
73	Female	56	Urban	Colorectal cancer	Negative	-	-
74	Female	63	Urban	Colorectal cancer	Negative	-	-
75	Female	68	Urban	Colorectal cancer	Negative	-	-

76	Female	62	Urban	Colorectal cancer	Negative	-	-
77	Female	63	Urban	Colorectal cancer	Negative	-	-
78	Female	60	Urban	Colorectal cancer	Negative	-	-
79	Female	47	Urban	Colorectal cancer	Negative	-	-
80	Female	66	Urban	Colorectal cancer	Negative	-	-
81	Female	69	Urban	Colorectal cancer	Negative	-	-
82	Female	65	Urban	Colorectal cancer	Negative	-	-
83	Female	48	Urban	Colorectal cancer	Negative	-	-
84	Female	48	Urban	Colorectal cancer	Negative	-	-
85	Female	53	Urban	Colorectal cancer	Negative	-	-
86	Female	67	Urban	Colorectal cancer	Negative	-	-
87	Female	63	Urban	Colorectal cancer	Negative	-	-
88	Female	48	Urban	Colorectal cancer	Negative	-	-
89	Female	59	Urban	Colorectal cancer	Negative	-	-
90	Female	74	Urban	Colorectal cancer	Negative	-	-
91	Female	51	Urban	Colorectal cancer	Negative	-	-
92	Female	63	Urban	Colorectal cancer	Negative	-	-
93	Female	52	Urban	Colorectal cancer	Negative	-	-
94	Female	64	Urban	Colorectal cancer	Negative	-	-
95	Female	64	Urban	Colorectal cancer	Negative	-	-
96	Female	65	Urban	Colorectal cancer	Negative	-	-
97	Female	66	Urban	Colorectal cancer	Negative	-	-
98	Female	63	Urban	Colorectal cancer	Negative	-	-
99	Female	68	Urban	Colorectal cancer	Negative	-	-
100	Female	70	Urban	Colorectal cancer	Negative	-	-
101	Female	74	Urban	Colorectal cancer	Negative	-	-

102	Female	61	Urban	Colorectal cancer	Negative	-	-
103	Female	36	Rural	Colorectal cancer	Negative	-	-
104	Female	50	Rural	Colorectal cancer	Negative	-	-
105	Female	61	Rural	Colorectal cancer	Negative	-	-
106	Female	65	Rural	Colorectal cancer	Negative	-	-
107	Female	48	Rural	Colorectal cancer	Negative	-	-
108	Female	41	Rural	Colorectal cancer	Negative	-	-
109	Female	69	Rural	Colorectal cancer	Negative	-	-
110	Female	44	Rural	Colorectal cancer	Negative	-	-
111	Female	70	Rural	Colorectal cancer	Negative	-	-
112	Female	45	Rural	Colorectal cancer	Negative	-	-
113	Female	47	Rural	Colorectal cancer	Negative	-	-
114	Female	53	Rural	Colorectal cancer	Negative	-	-
115	Female	83	Rural	Colorectal cancer	Negative	-	-
116	Female	65	Rural	Colorectal cancer	Negative	-	-
117	Male	78	Urban	Gastric cancer	Positive	*C. parvum*	IIaA15G2R1
118	Male	49	Urban	Gastric cancer	Positive	*C. parvum*	IIaA15G2R2
119	Male	56	Urban	Gastric cancer	Negative	-	-
120	Male	60	Urban	Gastric cancer	Negative	-	-
121	Male	72	Urban	Gastric cancer	Negative	-	-
122	Male	43	Urban	Gastric cancer	Negative	-	-
123	Male	57	Urban	Gastric cancer	Negative	-	-
124	Male	68	Urban	Gastric cancer	Negative	-	-
125	Male	55	Urban	Gastric cancer	Negative	-	-
126	Male	61	Urban	Gastric cancer	Negative	-	-
127	Male	79	Urban	Gastric cancer	Negative	-	-

128	Male	61	Urban	Gastric cancer	Negative	-	-
129	Male	31	Urban	Gastric cancer	Negative	-	-
130	Male	68	Urban	Gastric cancer	Negative	-	-
131	Male	63	Urban	Gastric cancer	Negative	-	-
132	Male	58	Urban	Gastric cancer	Negative	-	-
133	Male	58	Urban	Gastric cancer	Negative	-	-
134	Male	75	Rural	Gastric cancer	Negative	-	-
135	Male	48	Rural	Gastric cancer	Negative	-	-
136	Male	57	Rural	Gastric cancer	Negative	-	-
137	Male	59	Rural	Gastric cancer	Negative	-	-
138	Male	53	Rural	Gastric cancer	Negative	-	-
139	Male	64	Rural	Gastric cancer	Negative	-	-
140	Male	62	Rural	Gastric cancer	Negative	-	-
141	Male	64	Rural	Gastric cancer	Negative	-	-
142	Male	73	Rural	Gastric cancer	Negative	-	-
143	Male	67	Rural	Gastric cancer	Negative	-	-
144	Male	55	Rural	Gastric cancer	Negative	-	-
145	Male	60	Rural	Gastric cancer	Negative	-	-
146	Male	60	Rural	Gastric cancer	Negative	-	-
147	Male	65	Rural	Gastric cancer	Negative	-	-
148	Male	60	Rural	Gastric cancer	Negative	-	-
149	Male	63	Rural	Gastric cancer	Negative	-	-
150	Male	40	Rural	Gastric cancer	Negative	-	-
151	Male	45	Rural	Gastric cancer	Negative	-	-
152	Male	38	Rural	Gastric cancer	Negative	-	-
153	Male	64	Rural	Gastric cancer	Negative	-	-

154	Male	66	Rural	Gastric cancer	Negative	-	-
155	Female	44	Urban	Gastric cancer	Negative	-	-
156	Female	58	Urban	Gastric cancer	Negative	-	-
157	Female	53	Urban	Gastric cancer	Negative	-	-
158	Female	75	Urban	Gastric cancer	Negative	-	-
159	Female	87	Urban	Gastric cancer	Negative	-	-
160	Female	58	Urban	Gastric cancer	Negative	-	-
161	Female	56	Urban	Gastric cancer	Negative	-	-
162	Female	42	Urban	Gastric cancer	Negative	-	-
163	Female	54	Rural	Gastric cancer	Negative	-	-
164	Female	45	Rural	Gastric cancer	Negative	-	-
165	Female	42	Rural	Gastric cancer	Negative	-	-
166	Female	54	Rural	Gastric cancer	Negative	-	-
167	Female	80	Rural	Gastric cancer	Negative	-	-
168	Female	55	Rural	Esophageal cancer	Positive	*C. parvum*	IIaA15G2R2
172	Male	61	Urban	Esophageal cancer	Negative	-	-
173	Male	46	Urban	Esophageal cancer	Negative	-	-
174	Male	59	Urban	Esophageal cancer	Negative	-	-
178	Male	66	Urban	Esophageal cancer	Negative	-	-
179	Male	60	Urban	Esophageal cancer	Negative	-	-
180	Male	64	Urban	Esophageal cancer	Negative	-	-
181	Male	69	Urban	Esophageal cancer	Negative	-	-
169	Male	68	Rural	Esophageal cancer	Negative	-	-
170	Male	55	Rural	Esophageal cancer	Negative	-	-
171	Male	55	Rural	Esophageal cancer	Negative	-	-
176	Male	49	Rural	Esophageal cancer	Negative	-	-

177	Male	66	Rural	Esophageal cancer	Negative	-	-
182	Male	58	Rural	Esophageal cancer	Negative	-	-
175	Female	63	Rural	Esophageal cancer	Negative	-	-
183	Female	63	Rural	Esophageal cancer	Negative	-	-
184	Male	52	Urban	Liver cancer	Positive	*C. parvum*	IIaA15G2R2
185	Male	42	Urban	Liver cancer	Negative	-	-
186	Male	82	Urban	Liver cancer	Negative	-	-
187	Male	55	Urban	Liver cancer	Negative	-	-
188	Male	49	Rural	Liver cancer	Negative	-	-
189	Female	45	Rural	Liver cancer	Negative	-	-
190	Female	63	Rural	Liver cancer	Negative	-	-
191	Male	55	Urban	Small intestine cancer	Positive	*C. parvum*	IIaA15G2R2
192	Male	58	Urban	Small intestine cancer	Positive	*C. parvum*	IIaA15G2R1
193	Female	63	Urban	Small intestine cancer	Negative	-	-
194	Female	65	Urban	Small intestine cancer	Negative	-	-
195	Female	58	Urban	Small intestine cancer	Negative	-	-

Table S3. Frequencies of *Cryptosporidium* spp. in colorectal cancer patients by age, sex and residence (n=116).

Characteristic	No. examined	No. positive	%	X^2/*df*/*P-value*
**Age (years)**				
≤50	28	5	17.86	1.488/2/0.475
51-60	31	4	12.9	
>60	57	11	19.3	
**Sex**				
Male	67	15	22.39	5.357/1/0.021
Female	49	5	10.2	
**Residence**				
Urban	72	12	16.67	0.035/1/0.852
Rural	44	8	18.18	

Table S4. Information for the reference sequences used in this study.

GenBank ID	Isolate	Species	Genotype	Host	Product	Country
JX886765.1	CpB3	*C. parvum*	-	Cattle	small subunit ribosomal RNA	Turkey
JX886766.1	CpB4	*C. parvum*	-	Cattle	small subunit ribosomal RNA	Turkey
JX886767.1	CpB5	*C. parvum*	-	Cattle	small subunit ribosomal RNA	Turkey
JX886768.1	CpB6	*C. parvum*	-	Cattle	small subunit ribosomal RNA	Turkey
FJ752165.1	Tulare Dairy	*C. parvum*	-	Dairy calf	18S ribosomal RNA	USA
AY262034.1	2636	*C. parvum*	IIaA15G2R1	-	60 kDa glycoprotein	USA
DQ192501.1	9871	*C. parvum*	IIaA15G2R2	*Homo sapiens*	60 kDa glycoprotein	Canada
